# Risk Factor Analysis and Prediction of Severe Hypocalcemia after Total Parathyroidectomy without Auto-Transplantation in Patients with Secondary Hyperparathyroidism

**DOI:** 10.1155/2023/1901697

**Published:** 2023-01-16

**Authors:** Chenchen He, Yibing Zhang, Longfei Li, Guangming Cheng, Wei Zhang, Yufu Tang, Chunhui Wang

**Affiliations:** ^1^Department of Hepatobiliary and Thyroid Surgery, General Hospital of Northern Theater Command, Shenyang, China; ^2^China Medical University, Shenyang, China; ^3^Department of Medical Affairs, The General Hospital of Northern Theater Command, Shenyang, China

## Abstract

**Objective:**

Our study aimed to develop and validate a nomogram to predict severe hypocalcemia (SH) before total parathyroidectomy (TPTX) without auto-transplantation in patients with secondary hyperparathyroidism.

**Methods:**

A total of 299 consecutive patients who underwent TPTX without transplantation for secondary hyperparathyroidism were selected from the General Hospital of Northern Theater Command between January 2013 and December 2021. Of these, patients who underwent surgery between January 2013 and December 2020 formed the training cohort (*n* = 208) to develop a nomogram, and those who underwent surgery thereafter formed the validation cohort (*n* = 91) to validate the performance of this nomogram. Univariate and multivariate logistic regression analyses were used to identify the risk factors associated with SH, and then, a nomogram was constructed.

**Results:**

The incidence of postoperative SH was 27.9% and 35.2% in the training and validation cohorts, respectively. The preoperative factors associated with SH were younger age, lower serum calcium (Ca) level, higher intact parathyroid hormone (iPTH) level, and higher serum alkaline phosphatase (ALP) level. Incorporating these 4 factors, the nomogram achieved good concordance indexes of 0.866 (95%CI, 0.816–0.916) and 0.867 (95% CI, 0.793–0.941) in predicting SH in the training and validation cohorts, respectively, and had well-fitted calibration curves. The positive predictive values of the nomogram were 64.7% (54.1%–78.4%) and 75.0% (58.6%–88.5%), and negative predictive values of the nomogram were 90.0% (82.9%–93.6%) and 86.4% (73.5%–94.0%) for the training and validation cohorts, respectively.

**Conclusions:**

We developed and validated a nomogram for the prediction of SH in patients who underwent TPTX without auto-transplantation for secondary hyperparathyroidism. Our nomogram may facilitate the identification of high-risk SH in patients after TPTX and optimization of preoperative decision-making.

## 1. Introduction

Chronic kidney disease (CKD) is a global health problem with increasing prevalence [1, 2]. According to previous surveys, the prevalence of CKD was 11.7%–15.1% in China [2]. Secondary hyperparathyroidism (SHPT) is a common complication of CKD, and nearly all patients with end-stage renal disease (ESRD) will develop SHPT [3, 4]. Extensive evidence has shown that prolonged SHPT is closely correlated with high incidences of cardiovascular disease, bone fracture, and mortality [5–11]. Therefore, the control of SHPT in CKD is of utmost importance.

Surgical parathyroidectomy (PTX) is necessary for those patients with severe and progressive SHPT refractory to medical treatment [12]. Previous studies showed that PTX can relieve symptoms, improve quality of life, and reduce the risk of all-cause and cardiovascular mortality in patients with severe SHPT [13–17]. Postoperative hypocalcemia is the most common complication of surgery, and the incidence was up to 97% in a previous study [18]. Importantly, severe hypocalcemia (SH) can lead to life-threatening sequelae, such as respiratory muscle weakness, laryngeal stridor, seizures, cardiac arrhythmias, congestive heart failure, tetany, and even sudden death [19]. Therefore, an accurate preoperative prediction of SH can help doctors make effective response and avoid the occurrence of adverse events. The aim of this study was to investigate the risk factors of postoperative SH following total PTX (TPTX), and a nomogram was constructed to predict the development of postoperative SH using preoperative clinical characteristics in patients with SHPT who had undergone TPTX.

## 2. Materials and Methods

### 2.1. Patients

Between January 2013 and December 2021, data on consecutive patients with SHPT who had undergone TPTX in the General Hospital of Northern Theater Command were obtained. The study was approved by the Institutional Ethics Committee of the General Hospital of Northern Theater Command. All patients were informed of the risks and procedures of the surgery and signed informed consent.

The inclusion criteria were as follows: (1) in accordance with the Kidney Disease Outcomes Quality Initiative guidelines, patients with persistently elevated serum intact PTH (iPTH) levels >800 pg/ml, uncontrolled hypercalcemia with hyperphosphatemia, severe clinical symptoms such as bone and joint pain, muscle weakness, or refractory pruritus, or refractory to medical treatment, need to undergo PTX; (2) patients underwent total PTX without auto-transplantation; (3) the surgery is technically successful with the pathological confirmation of at least 4 parathyroid glands, accompanied by an intact parathormone (iPTH) value of <60  pg/mL on postoperative day 1 (POD1). Patients who underwent TPTX with auto-transplantation or subtotal PTX (SPTX), underwent second PTX due to recurrent SHPT following the initial PTX, underwent a failure operation, had a history of liver, biliary, or pancreatic diseases, and had incomplete clinical data were excluded. Patients who underwent surgery between January 2013 and December 2020 were included in the training cohort for development of the nomogram, and those who underwent surgery between January 2021 between December 2021 were included in the validation cohort.

### 2.2. Clinical Variables

We collected preoperative information on clinical variables, including gender, age, body mass index (BMI), underlying diseases, dialysis duration, dialysis modality, and preoperative laboratory tests (serum intact parathyroid hormone, serum alkaline phosphatase, serum calcium, serum phosphate, serum kalium, hemoglobin, albumin, serum creatinine, urea, prothrombin, and fibrinogen). Moreover, the postoperative serum calcium within 72 hours had been collected in this study. The postoperative serum calcium below 2.20 mmol/L was used to diagnose hypocalcemia, and severe hypocalcemia was defined as serum calcium below 1.80 mmol/L after TPTX.

## 3. Perioperative Management and Surgical Procedures

Routine preoperative examination included serum intact PTH level, concentrations of calcium, blood phosphorus, liver and renal function tests, and ultrasonography of the thyroid and parathyroid glands. The preoperative diagnosis was based on criteria of the KDIGO 2009 clinical practice guideline. TPTX with/without auto-transplantation of parathyroid tissue and SPTX are currently considered as standard surgical procedures in the treatment of SHPT [20, 21]. Previous studies have noted that the TPTX without auto-transplantation approach has been associated with lower rates of recurrence [22]. Therefore, total parathyroidectomy without auto-transplantation is used as a surgical option in our center. TPTX with auto-transplantation (TPTX + AT) and SPTX had not been selected since January 2016 in our center. All surgical procedures were performed by Dr. Guangming Cheng and his surgical team. A successful operation was defined as previously described [23].

### 3.1. Statistical Analysis

Continuous variables were expressed as the means and standard deviations or medians and interquartile ranges (IQRs) as appropriate. Categorical variables were summarized as the counts and percentages in each category. The related variables were compared using Student's *t*-test, Mann–Whitney *U*, Chi-squared test, or Fisher exact test. Univariable logistic analysis was used to identify clinically relevant variables associated with postoperative hypocalcemia in the training cohort. All variables associated with hypocalcemia at a significant level were candidates for multivariate logistic analysis. A nomogram was formulated based on the results of multivariate logistic regression analysis and by using VRPM package of R version 4.1.3 (http://mirror.bjtu.edu.cn/cran/bin/windows/base/). The predictive performance of the nomogram was measured with the concordance index (C index) and calibration with 1000 bootstrap samples to decrease the overfit bias. All statistical analyses were performed by using the R software studio (version 4.1.3), and a *P* value of less than 0.05 was considered to be statistically significant [24].

## 4. Results

### 4.1. Patients' Characteristics

From January 2013 to December 2021, 403 consecutive patients who had secondary hyperparathyroidism underwent parathyroidectomy. Of these, 299 patients who met the inclusion criteria were enrolled, and 208 and 91 patients were divided into the training and validation cohorts, respectively. The clinical characteristics of the patients are listed in Table 1. The baseline clinical data were similar between the training and validation cohorts. The postoperative SH was found in 27.9% and 35.2% patients in the 2 cohorts, respectively.

### 4.2. Prediction of Postoperative Severe Hypocalcemia

Only preoperative variables were used in this analysis. The univariate analyses revealed that younger age (OR = 0.929, 95% CI: 0.903–0.957, *P* < 0.001), lower hemoglobin level (OR = 0.976, 95% CI: 0.959–0.993, *P*=0.006), lower preoperative serum calcium level (OR = 0.025, 95% CI: 0.005–0.118, *P* < 0.001), higher preoperative serum iPTH (OR = 2.250, 95% CI: 1.789–15.403, *P*=0.003), and higher preoperative serum ALP level (OR = 7.893, 95% CI: 3.709–16.797, *P* < 0.001) were significantly associated with postoperative SH (Table 2). All of the aforementioned significant parameters were then included in multivariate logistic regression analysis. The results showed that younger age (OR = 0.942, 95% CI: 0.908–0.976, *P*=0.001), lower preoperative serum calcium level (OR = 0.026, 95% CI: 0.004–0.193, *P* < 0.001), higher preoperative serum iPTH (OR = 5.864, 95% CI: 1.499–22.938, *P*=0.011), and higher preoperative serum ALP level (OR = 3.144, 95% CI: 1.276–7.745, *P*=0.013) were independent risk factors associated with postoperative SH (Table 3).

### 4.3. Nomogram for Predicting Postoperative Severe Hypocalcemia

**Figure 1:**
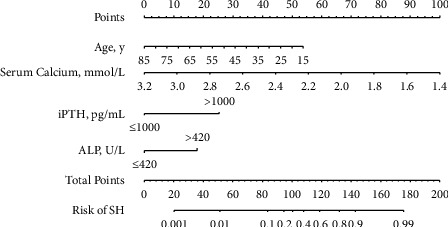
Nomogram for preoperative prediction of server hypocalcemia (SH) following total parathyroidectomy (TPTX) without auto-transplantation in patients with secondary hyperparathyroidism (SHPT). Points are signed for age, preoperative serum calcium level, preoperative serum iPTH level, and preoperative serum ALP level. The score for each value was assigned by drawing a line upward to the “points” line, and the sum of the four scores was plotted on the “total points” line (probability of SH).

**Figure 2:**
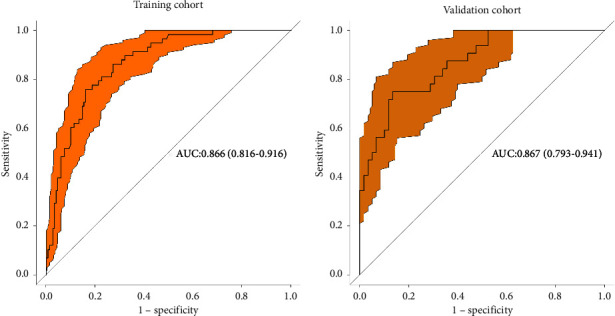
Receiver operating characteristics (ROC) of the nomogram in the training cohort (a) and validation cohort (b).

**Figure 3:**
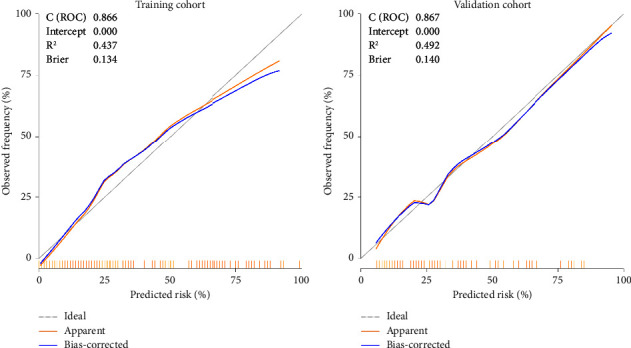
Validity of the predictive performance of the nomogram in estimating the risk of SH presence in the training cohort (*n* = 208) and in the validation cohort (*n* = 91).

Based on the previous analyses, the independently associated risk factors were used to construct a nomogram (Figure 1). The resulting model was internally validated using the bootstrap validation method. The nomogram for predicting postoperative SH in the training cohort had an unadjusted *C* index of 0.866 (95%CI, 0.816–0.916) and a bootstrap-corrected *C* index of 0.866, indicating that the nomogram has good accuracy in estimating the risk of SH. In the validation cohort, the nomogram displayed a *C* index of 0.867 (0.793–0.941) for the estimation of SH risk. There was also a good calibration curve for risk estimation (Figure 2). In addition, the calibration plots overlapped with the ideal line in the training and validation cohorts, showing adequate agreement of the predictive nomogram with actual observations (Figure 3).

### 4.4. Risk of Postoperative Severe Hypocalcemia Based on the Nomogram

The optimal cutoff value of the total nomogram scores was determined to be 100. The sensitivity, specificity, positive predictive value, and negative predictive value when used in differentiating the presence from absence of SH were 75.9%, 84.0%, 64.7%, and 90.0% in the training cohort and 75.0%, 86.4%, 75.0%, and 86.4% in the validation cohort, respectively (Table 4).

## 5. Discussion

Postoperative SH can increase mortality and hospitalization [25–28]. In the present study, SH was found to be present in 90 of 299 cases. And, we uncovered that the preoperative factors, including younger age, higher serum iPTH level, higher serum ALP level, and lower serum calcium level, are significantly associated with SH in patients who underwent TPTX without auto-transplantation. Importantly, we developed a nomogram which achieved an optimal preoperative prediction of SH in those patients who underwent TPTX without auto-transplantation for secondary hyperparathyroidism.

Considering the high incidence of SH after parathyroidectomy and the development of life-threatening sequelae, efforts on the risk estimation of SH have been made over the past decade [29–46]. Various risk factors have been suggested for the development of postoperative hypocalcemia in previous studies, including younger age, pruritus, higher preoperative iPTH and ALP levels, and lower preoperative serum calcium level. Moreover, the diagnostic value of these factors was evaluated by using receiver operator characteristic (ROC) analyses [31, 33, 40, 41]. For example, one study reported a diagnostic model incorporated 3 factors (preoperative serum calcium, iPTH, and ALP levels) in the risk estimation of SH [40]. However, the algorithm of this diagnostic model is complex, and further clinical validation is required. As is well known, the nomogram, identified as an easy-to-use prediction tool, has high accuracy and good discrimination characteristics in predicting outcomes. In the current study, the proposed nomogram, which incorporated 4 easily available preoperative variables, performed well as supported by the *C* index values 0.866 (95%CI, 0.816–0.916) and 0.867 (95% CI, 0.793–0.941) in the training and validation cohorts, respectively, and the optimal calibration curves demonstrated the agreement between prediction and actual observation.

Younger age was found to be a preoperative risk factor for SH in this study, which is largely consistent with the previous reports [30, 34–36, 43, 44, 46]. Explanation as to why younger patients are at a higher risk of hypocalcemia mainly includes stronger osteoblast function and greater calcium utilization efficiency of bone tissue. However, the influence of age on the postoperative hypocalcemia is still controversial. Gong et al. reported that advanced age is a risk factor of postoperative hypocalcemia [39]. In addition, some reports also revealed no association between age and postoperative hypocalcemia. It should be noticed that nearly all of these studies had been performed in a single center with a small sample size. Therefore, some researchers have appealed for more studies with larger sample sizes to verify this conclusion. Recently, in a retrospective study that included 1500 patients, Zhao et al. reported that age at the time of surgery was negatively associated with postoperative hypocalcemia [43]. The sample size of this retrospective study is larger than that in other previous studies and seems to confirm the association between younger age and postoperative hypocalcemia.

The explanation of preoperative serum calcium level as a risk factor of hypocalcemia after parathyroid surgery is limited in the relevant studies [11, 33, 35, 36, 46]. The lower preoperative serum calcium in SHPT patients may indicate a higher baseline bone-remodeling status [47]. And, the remineralization of the skeleton would be stronger in those patients after PTX. Therefore, the frequency and the severity of postoperative hypocalcemia would be higher in those patients. Some studies use preoperative corrected serum calcium for research [35]. However, corrected serum calcium cannot reflect the accurate serum calcium concentration in patients [48–50]. For patients undergoing long-term dialysis or patients with hypoproteinemia, the blood calcium level may be overestimated when using corrected serum calcium and the collinearity among variables is often neglected in these studies when screening variables. In the present study, total serum calcium level was used to predict SH after TPTX, and we identified that calcium deficiency before operation was a risk factor for SH. Importantly, the results of this study strongly suggest that appropriate calcium supplement therapy should be provided in patients with hypocalcemia before PTX to alleviate the postoperative complications of hypocalcemia.

In previous studies, preoperative iPTH as one of the risk factors of postoperative hypocalcemia has been frequently found in patients who underwent PTX [29, 31–34, 36, 37, 39, 40, 43, 44]. It is well accepted that preoperative iPTH concentration can be used to predict postoperative hypocalcemia, because it is consistent with the physiological functions of iPTH. However, some studies also found no association between preoperative iPTH and postoperative hypocalcemia. It should be noticed that the parathyroid procedures performed are rather variable in these studies [30, 33, 38, 41, 42, 46], including TPTX, TPTX + AT, or SPTX. The selected operative method definitely affects the postoperative serum calcium content. For example, SPTX preserves a remnant parathyroid gland with its original blood supply, and then, it has a higher iPTH value and a lower risk of postoperative hypocalcemia [51, 52]. Therefore, the results of these studies could not truly reflect the association between preoperative iPTH and postoperative hypocalcemia. In addition, there are other explanations including skeletal resistance to iPTH, the relationship between the serum iPTH level and degree of bone remodeling being not always maintained, and the current iPTH assay being not accurate [53–55].

ALP as a bone formation biomarker can reflect the activity of osteoblasts [56]. The serum ALP level will be increased when the osteoblastic activity is stimulated by high level of iPTH in patients with SHPT. Theoretically, higher ALP levels before surgery indicates a more active bone remodeling state in patients, resulting in a higher incidence of hypocalcemia after PTX. In fact, preoperative ALP level as a risk factor of hypocalcemia after PTX has been well identified in previous studies [29–32, 34, 36–45]. For example, a previous study conducted by Tan et al. reported that preoperative ALP level was a risk factor of hypocalcemia, and it suggested that preoperative ALP level should be used to instruct the management of postoperative hypocalcemia [45]. Ho et al. also identified that preoperative ALP level was a risk factor for postoperative hypocalcemia, and they considered that the increase in ALP level was closely related to the decrease in serum calcium level and the amount of calcium supplement by observing the clinical index two weeks after operation [30]. Bone-ALP, which is directly related to bone turn-over, had been used in several previous studies [35]. But, bone-ALP is not routinely measured in most of the the institutions especially in the basic medical institutions, thus limiting its wide use. In the present study, we selected total ALP but not bone-ALP as a variable, and we found that total preoperative ALP level is an independent predictive factor of hypocalcemia after TPTX.

Moreover, the accuracy of this nomogram was estimated using as the cutoff value in the present study. Patients with a score 100 or more are considered a high-risk subgroup of SH after TPTX. Our results identified that this nomogram allows physicians to accurately identify dialysis patients who are at a greater risk of hypocalcemia after PTX and to aggressively monitor and treat those patients with a score or more.

There are some limitations in our study. First and foremost, we took the first measurement of blood level of calcium at 6–8 hours after surgery and then once daily in the morning until 72 hours after surgery. We choose the lowest value of them to distinguish the sever hypocalcemia. Hypocalcemia could start as early as 5-6 hours, and patients were treated with intravenous infusion of calcium gluconate if they were diagnosed as hypocalcemia regardless of the severity. However, the use of calcium supplements can lead to decreased incidence and severity of hypocalcemia. Second, the nomogram was constructed based on data from a single institution. It is necessary to validate the predictive value of this nomogram in the other institutions. Third, some other factors might be correlated with postoperative hypocalcemia, such as uremic toxins and bone mineral density. Finally, this study is retrospective in nature. The reliability of the nomogram model needs to be confirmed in further prospective studies.

## 6. Conclusion

In the present study, we identified younger age, higher serum iPTH level, higher serum ALP level, and lower serum calcium level are the preoperative risk factors for SH after TPTX. By combining these 4 preoperative risk factors, a nomogram was constructed. The nomogram provides an optimal preoperative estimation of SH risk in patients with SHPT underwent TPTX.

## Figures and Tables

**Table 1:** Clinical characteristics of 299 patients undergoing TPTX.

Variable	Cohort, no. (%)		
	Training *N* = 208	Validation *N* = 91	*P* value
Gender			0.122
Male	97 (46.6)	52 (57.1)	
Female	111 (53.4)	39 (42.9)	
Age, median (IQR) (y)	49.0 (35.0–56.0)	46.0 (38.0–56.0)	0.898
BMI (kg/m^2^)			0.179
≤25	165 (79.3)	65 (71.4)	
>25	43 (20.7)	26 (28.6)	
Underlying diseases			0.053
Glomerulonephritis	51 (24.5)	23 (25.2)	
Diabetic nephropathy	8 (3.9)	3 (3.3)	
Hypertensive nephropathy	11 (5.3)	10 (11.0)	
Polycystic kidney	10 (4.8)	6 (6.6)	
Others	21 (10.1)	17 (18.7)	
Unknown	107 (51.4)	32 (35.2)	
Duration of dialysis, median (IQR) (y)	8.00 (6.00–11.0)	8.00 (6.00–10.0)	0.928
Dialysis modality			0.055
Hemodialysis	199 (95.7)	81 (89.0)	
Peritoneal dialysis	9 (4.3)	10 (11.0)	
iPTH (pg/ml)			0.292
≤1000	46 (22.1)	26 (28.6)	
>1000	162 (77.9)	65 (71.4)	
Alkaline phosphatase (U/L)			0.697
≤420	168 (80.8)	71 (78.0)	
>420	40 (19.2)	20 (22.0)	
Serum calcium, median (IQR) (mmol/L)	2.47 (2.31–2.60)	2.44 (2.29–2.59)	0.342
Serum phosphate, mean (SD) (mmol/L)	2.37 (0.55)	2.45 (0.55)	0.264
Serum kalium, mean (SD) (mmol/L)	4.74 (0.69)	4.57 (0.71)	0.053
Hemoglobin, mean (SD) (g/L)	106 (18.4)	109 (19.6)	0.281
Albumin, median (IQR) (g/L)	39.0 (35.9–41.0)	38.4 (35.1–41.0)	0.454
Serum creatinine, median (IQR) (*μ*mol/L)	904 (748–1080)	993 (790–1168)	0.125
Blood urea nitrogen, median (IQR) (mmol/L)	21.5 (16.9–26.4)	19.0 (14.8–26.0)	0.073
Prothrombin time, median (IQR) (s)	13.3 (12.8, 13.9)	13.5 (13.1, 13.9)	0.075
Fibrinogen, median (IQR), (g/L)	4.20 (3.56–4.99)	4.32 (3.73–5.14)	0.181
Severe hypocalcemia			0.260
No	150 (72.1)	59 (64.8)	
Yes	58 (27.9)	32 (35.2)	

BMI: body mass index; iPTH: intact parathyroid hormone; IQR: interquartile range; SD: standard deviation.

**Table 2:** Univariate logistic regression analysis of SH presence based on preoperative data in the training cohort.

Variable	OR (95%CI)	*P* value
Gender, male vs female	1.461 (0.795–2.685)	0.222
Age (y)	0.929 (0.903–0.957)	<0.001
BMI, >25 vs ≤25 (kg/m^2^)	1.328 (0.629–2.742)	0.444
Underlying diseases		
Diabetic nephropathy vs glomerulonephritis	0.418 (0.021–2.676)	0.434
Hypertensive nephropathy vs glomerulonephritis	2.436 (0.612–9.477)	0.194
Polycystic kidney vs glomerulonephritis	0.731 (0.102–3.397)	0.713
Others vs glomerulonephritis	1.462 (0.468–4.371)	0.501
Unknown vs glomerulonephritis	1.139 (0.541–2.488)	0.737
Duration of dialysis (y)	0.935 (0.858–1.021)	0.133
Dialysis modality, peritoneal dialysis vs hemodialysis	3.443 (0.880–14.369)	0.073
iPTH, >1000 vs ≤1000 (pg/mL)	2.250 (1.789–15.403)	0.003
Alkaline phosphatase, >420 vs ≤420 (U/L)	7.893 (3.709–16.797)	<0.001
Serum calcium (mmol/L)	0.025 (0.005–0.118)	<0.001
Serum phosphate (mmol/L)	1.389 (0.800–2.411)	0.243
Serum kalium (mmol/L)	0.765 (0.491–1.191)	0.236
Hemoglobin (g/L)	0.976 (0.959–0.993)	0.006
Albumin (g/L)	0.902 (0.834–0.975)	0.009
Serum creatinine (*μ*mol/L)	1.000 (0.999–1.002)	1.000
Blood urea nitrogen (mmol/L)	1.018 (0.972–1.066)	0.449
Prothrombin time (s)	1.629 (1.098–2.417)	0.015
Fibrinogen (g/L)	1.023 (0.923–1.134)	0.666

BMI: body mass index; iPTH: intact parathyroid hormone; OR: odds ratio; CI: confidence interval.

**Table 3:** Multivariate logistic regression analysis of SH presence based on preoperative data in the training cohort.

Variable	*β*	OR (95%CI)	*P* value
Age (y)	−0.060	0.942 (0.908–0.976)	0.001
iPTH (pg/mL), >1000 vs ≤1000	1.769	5.864(1.499–22.938)	0.011
Alkaline phosphatase (U/L) >420 vs ≤420	1.145	3.144 (1.276–7.745)	0.013
Serum calcium (mmol/L)	−3.631	0.026 (0.004–0.193)	<0.001
Hemoglobin (g/L)	−0.009	0.991 (0.968–1.014)	0.444
Albumin (g/L)	−0.048	0.953 (0.854–1.064)	0.394
Prothrombin time (s)	0.167	1.181 (0.724–1.927)	0.505

iPTH: intact parathyroid hormone; OR: odds ratio; CI: confidence interval.

**Table 4:** Accuracy of the prediction score of the nomogram for estimating the risk of SH presence.

Variable	Value (95%CI)	
	Training cohort	Validation cohort
Area under ROC curve	0.866 (0.816–0.916)	0.867 (0.793–0.941)
Cut-off score	100	100
Sensitivity (%)	75.9 (62.8–86.1)	75.0 (56.6–88.5)
Specificity (%)	84.0 (77.1–89.5)	86.4 (75.0–94.0)
Positive predictive value (%)	64.7 (54.1–78.4)	75.0 (58.6–88.5)
Negative predictive value (%)	90.0 (82.9–93.6)	86.4 (73.5–94.0)
Positive likelihood ratio	4.741 (3.196–7.034)	5.531 (2.817–10.859)
Negative likelihood ratio	0.287 (0.181–0.456)	0.289 (0.157–0.532)

## Data Availability

The data that support the findings of this study are available from the corresponding authors upon reasonable request.
